# Early strategy of scepter XC balloon angioplasty and simultaneous Nimodipine infusion for vasospasm following ruptured aneurysm

**DOI:** 10.1186/s12883-020-01856-4

**Published:** 2020-07-07

**Authors:** Chun-Ting Chen, Ching-Chang Chen, Alvin Yi-Chou Wang, Yi-Ming Wu, Shy-Chyi Chin, Po-Chuan Hsieh, Mun-Chun Yeap, Shih-Yuan Hsu, Ya-Jui Lin

**Affiliations:** 1 Department of Neurosurgery, Stroke Center & Neurointervention, Chang Gung Memorial Hospital, Chang Gung Medical Center and University, 5, Fu-Shin Street, Kwei-Shan Hsiang, Taoyuan, 333 Taiwan; 2 Department of Neurosurgery, Guangdong Provincial Hospital of Chinese Medicine, Guangzhou, Guangdong China; 3 Department of Radiology, Division of Neuroradiology, Linkou Chang Gung Memorial Hospital & Chang Gung University, Taoyuan City, Taiwan; 4 Department of Neurosurgery, Kaohsiung Chang Gung Memorial Hospital, Kaohsiung City, Taiwan

**Keywords:** Balloon angioplasty; subarachnoid hemorrhage, Cerebral aneurysm, Aneurysm rupture, Scepter XC balloon

## Abstract

**Background:**

Cerebral vasospasm still results in high morbidity and mortality rates in patients after aneurysmal subarachnoid hemorrhage (SAH). The aim of this study was to establish a protocol for the management of vasospasm and demonstrate our experience of angioplasty using the Scepter XC balloon catheter.

**Methods:**

In this retrospective study, a computed tomography angiography and perfusion image was arranged if early symptoms occurred or on the 7th day following aneurysmal SAH. In patients with clear consciousness, balloon angioplasties were performed for symptomatic vasospasms, which were not improved within 6–12 h after maximal medical treatments. In unconscious patients, balloon angioplasties were performed for all patients with angiographic vasospasms.

**Results:**

Fifty patients underwent Scepter XC balloon angioplasty among 396 consecutive patients who accepted endovascular or surgical treatments for ruptured aneurysms. All angioplasty procedures were successful without complications. 100% angiographic improvement and 94% clinical improvement were reached immediately after the angioplasties. A favorable functional outcome (modified Rankin Score of ≤2) could be achieved in 82% of patients. Even in patients with poor clinical grading (Hunt–Hess grade 4–5), a clinical improvement rate of 87.5% and favorable outcome rate was 70.8% could be achieved.

**Conclusion:**

Balloon angioplasty with Scepter XC balloon catheter is safe and effective for post-SAH vasospasm. This device’s extra-compliant characteristics could considerably improve the quality of angioplasty procedures. For all patients, even those with poor neurological status, early treatment with combined protocol of nimodipine and angioplasty can have good clinical outcomes.

## Background

Cerebral vasospasm remains a major cause of delayed cerebral ischemia for patients following subarachnoid hemorrhage (SAH) from ruptured intracranial aneurysms. Of patients with aneurysmal SAH, 30 to 70% develop cerebral angiographic vasospasm, with delayed neurologic deficits manifesting in 30 to 50% of patients [1–3]. Noninvasive management approaches, including induction of hypertension, maintenance of euvolemia, and infusion of oral form or intravenous vasodilators, are the most common strategies [3–6]. However, vasospasm still results in mortality rates of 7 to 20% or permanent disability [1, 6, 7]. Balloon angioplasty is a more invasive procedure that can lead to an immediate and satisfying angiographic result in proximal vasospasm [8, 9]. However, it is usually postponed and reserved for when symptoms are refractory because of a potential risk of thromboembolic complications and even vessel rupture [10–12]. The Scepter XC balloon (4x11mm; Microvention, Tustin, CA, USA) is a user-friendly temporary consisting of a dual coaxial lumen attached to a low-inflation pressure-compliant balloon. It has excellent trackability and stability, and the extra-compliant design represents technical advancements in endovascular treatment [13–16]. Few studies have reported on the feasibility of the Scepter balloon for vasospasm treatment [9, 13, 14, 17]. In this study, we detailed the largest single-center case experience of Scepter XC balloon angioplasty and provided an early protocol that combined with simultaneously intra-arterial (IA) nimodipine infusion to treat vasospasm after aneurysm rupture.

## Methods

### Management of post-SAH vasospasm

Between January 2014 and January 2018, 396 consecutive patients accepted endovascular or surgical treatments for ruptured aneurysms in our institution. After securing the aneurysms, patients were sent to the neurosurgical intensive care unit. Standard medical management for vasospasm was continued, including nimodipine usage, fluid infusion to maintain euvolemic status, blood pressure control, and intensive monitoring. Clinically, vasospasm was suspected when new neurological deficits occurred. However, in unconscious patients, the new neurologic deficits were difficult to identify and the vasospasm might happen very early since aneurysm ruptured. We used bedside transcranial Doppler patients after aneurysmal SAH. However, transcranial Doppler was limited by lower sensitivity and poor acoustic window in some patients [18, 19]. If clinical or ultrasound diagnosis leaded to early detection of abnormalities, a computed tomography angiography (CTA) and perfusion (CTP) study would be arranged. If not, CTA/CTP would be routinely arranged on the 7th day following aneurysmal SAH to evaluate the level of vasospasm, brain perfusion, and to eliminate a diagnosis of hydrocephalus or re-hemorrhage. The high accuracy and sensitivity of CTA/CTP were well published and established in the literature [18, 20, 21]. All CTA/CTP images were critically reported and reviewed by two experienced neuroradiologists.

**Fig. 1:**
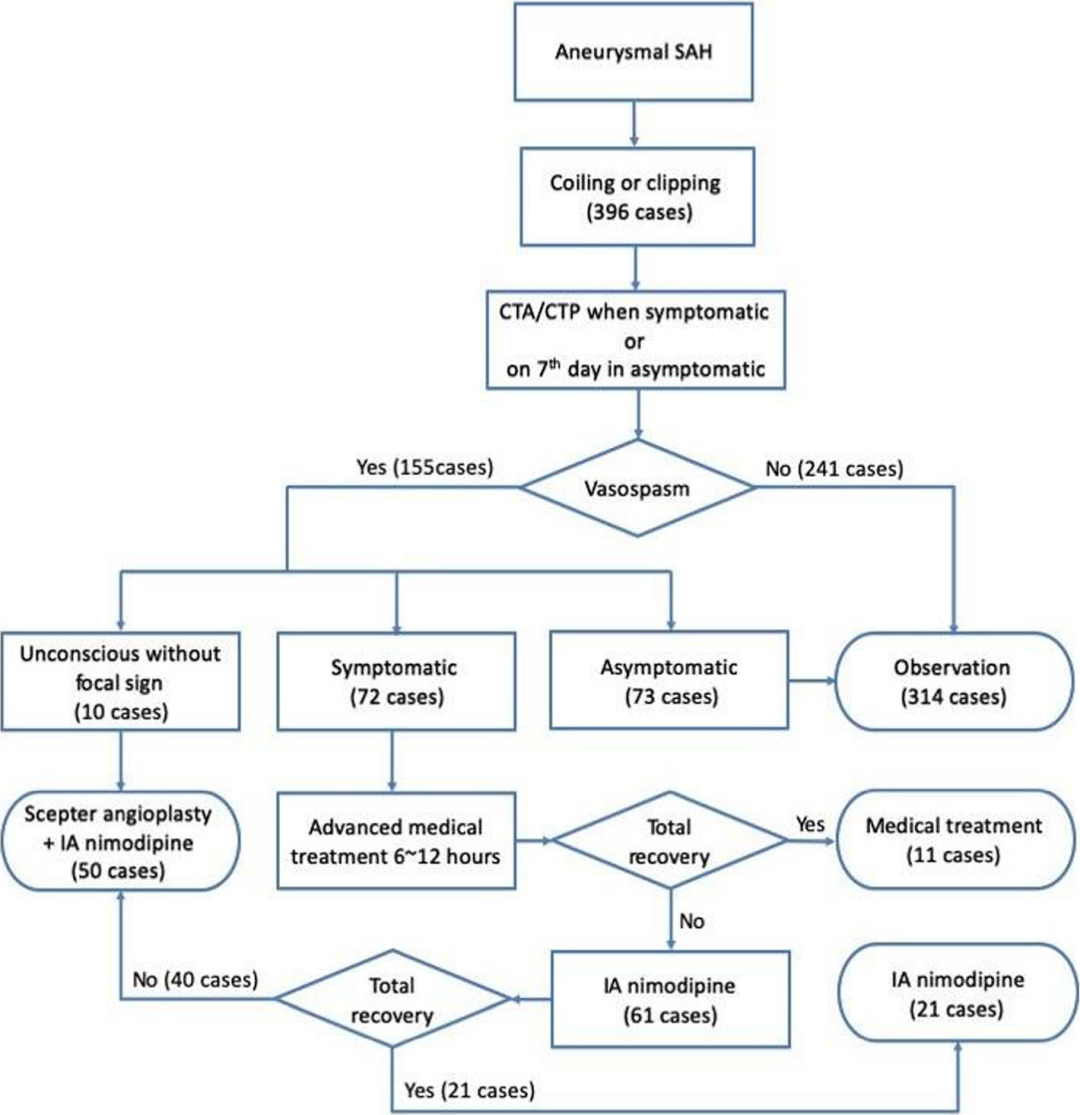
Flowchart of treatment for patients with post-SAH vasospasms in our institution

Hyperdynamic therapy was then be maximized to the point of elevating the mean arterial pressure by 15–20% and central venous pressure higher than 8-10cmH20 [8]. If the patients did not demonstrate neurological reversal totally within 6–12 h, they were transferred to the endovascular suite for angiography. In the patients with clear consciousness, endovascular treatments (Scepter XC balloon angioplasty and IA nimodipine) were administered for symptomatic vasospasms regarded as refractory to medical treatment. In unconscious patients, because their symptoms were difficult to recognize, balloon angioplasties were performed for all patients with radiologic vasospasms. The angiographic improvement was estimated immediately after post-angioplasty DSA and was compared with the routine pre-angioplasty DSA or baseline images before aneurysm treatment. Angiography improvement was defined as at least 30% improvement of vessels diameter after angioplasty; symptoms improvement were defined any clinical improvements of conscious level, muscle power, cognition, or speech. The protocol and treatment strategy for patients with post-SAH vasospasm are represented in Fig. 1.

Before angiography, vessel conditions for balloon angioplasty were analyzed, and a DSA was arranged to confirm the location and degree of vasospasms. For patients without larger vessel spasms or with only mild larger vessel spasm (< 25% stenosis) [9, 12], simple IA nimodipine infusions were arranged without angioplasty. The nimodipine infusion would continue at least 20–60 min depending on the angiographic improvement. If the patient’s symptoms did not recover, the procedure would be repeated again everyday for 1–3 days. If the angiographic improvement was not obvious after simple nimodipine or re-stenosis occurred, scepter balloon angioplasty will be performed. By this paradigm, 50 patients underwent Scepter XC balloon angioplasty from 2014 to 2018. Endovascular procedures including coils embolization and balloon angioplasty were performed by 3 neurosurgeons and 2 neurointerventionalists. Surgical clipping was performed by 7 neurosurgeons. All of them were coauthors and had over 5-years’ experience performing clipping or embolization. We retrospectively reviewed clinical, radiological, and endovascular findings for these patients, and the correlation between clinical characteristics and functional outcomes were analyzed. Good functional outcome was defined as modified Rankin Score (mRS) < =2, followed at least 3 months and recorded by the above surgeons in the outpatient department. Baseline characteristics and good outcome were compared using Chi-Square test. A *P* value < 0.05 was considered statistically significant. No adjustment of multiple testing (multiplicity) was made in this study. Data analyses were conducted using SPSS 25 (IBM SPSS Inc., Chicago, Illinois). This study was approved by the institutional review board (201800342B0).

### Procedure of scepter XC balloon angioplasty

All interventions were performed under general anesthesia. Initial DSA was performed to reveal the cerebral vasculature. Prior to cerebral angioplasty being performed, a bolus (6000 IU) of heparin was given after the sheath was introduced. A 6 Fr. guiding catheter with a pressure line (50 mg nimodipine in 1000 mL of normal saline solution at a rate of 1 to 2 mL/minute) was navigated to the vessel with the most severe vasospasm. After the guiding catheter reached the target vessel, the dripping speed of the IA nimodipine line was adjusted to 4 to 5 mL/minute. Blood pressure was continuously monitored via the arterial line throughout the procedure. If a decrease in blood pressure occurred, the anesthesiologist administered a dopamine infusion to maintain systolic pressure at 100 to 120 mmHg.

A Scepter XC balloon catheter (4x11mm; Microvention, Inc., Tustin, California, USA) was navigated to the most distal part of the vasospastic segment with which the operator was comfortable, such as anterior cerebral artery segment 2 (A2) or middle cerebral artery segment 3 (M3), under the assistance of a 0.014-in. Traxcess microwire (Microvention, Tustin, California, USA). The Scepter XC balloon was then inflated with gentle pressure with the lumen of the balloon not exceeding 60 to 70% of the normal diameter of the diseased vasculature. Once the balloon reached the desired diameter, it was deflated immediately. Because it takes less than 10 s to inflate and deflate the balloon in the diseased segment, prolonged inflation was not necessary. After the vasospastic distal segment was dilated, the balloon was moved proximally to dilate the residual vasospastic vessels. Occasionally, when the vasospasm was so severe that navigation of the balloon catheter was not possible, and if the proximal vessels were not dilated, angioplasty from the proximal to distal would be performed. During the angioplasty procedure, the pressurized nimodipine drip was maintained at 4 to 5 mL/minute. Balloon angioplasties were performed by experienced neurointerventionalists and the whole procedure was usually completed within 1 hour.

## Results

Between January 2014 and January 2018, 396 consecutive patients accepted endovascular or surgical treatments (clipping) for ruptured aneurysms in our institution. Among the 396 patients, 155 (39.14%) had post-SAH vasospasms found during routine imaging and only 72 patients (18.19%) had symptomatic vasospasms. After 6 to 12 h advanced medical treatment, 61 cases (15.4%) still had symptoms caused by vasospasm and underwent endovascular procedures. Twenty-one out of 61 patients, with no obvious large vessels spasms nor > 25% stenosis, exhibited improved symptoms after simple endovascular IA nimodipine infusion (between 1 and 3 sessions). The remaining 40 patients underwent balloon angioplasty. Ten patients, who had poor consciousness after operations, presented diffused severe vasospasm in the images study without obvious focal neurologic deficits. (Fig. 1).

A total of 50 patients with a mean age of 50 (range 28–68 years, 38 women and 12 men) underwent Scepter XC balloon angioplasties and were enrolled in this study. The clinical and angiographic characteristics and various results for the 50 patients are summarized in Table 1. Twenty-six patients (62%, 26/50) had a low clinical grade (Hunt–Hess grade 1–3; ten patients were grade 3), and 24 patients (48%, 24/50) had a high clinical grade (Hunt–Hess grade 4–5). Fifteen patients (30%) had Fisher grade 1–2 bleeding pattern, and 35 patients (70%) had Fisher grade 3–4 hemorrhagic pattern. The most commonly treated vessels were middle cerebral artery (MCA) M1 segments (94%, 47/50), followed by M2 segments (70%, 35/50), distal internal carotid arteries (ICA) (50%, 25/50), anterior cerebral arteries (ACA) (16%, 8/50), and vertebral arteries (VA) (14%, 7/50). In four cases, the balloon could reach proximal M3 branches for successful angioplasties. All angioplasty procedures were successful without any vessels rupture, dissection, or thromboembolic complications. The severity of vasospasm improved immediately and significantly (100%) after angiography. 94% (47/50) of patients exhibited symptom improvement within 24 h. Only one patient required secondary angiography for symptomatic vasospasm 3 days later on the opposite side, which exhibited no obvious vasospasm in the first angiography. Even in patients with a high clinical grading and poor hemorrhagic pattern, the immediate rate of improvement also reached approximately 90% (87.5 and 91.4%, respectively). Two of the patients in this study died. One death was resulted from sustained cerebral ischemia, brain swelling, and uncal herniation. The other patient who died had intractable status epilepticus with respiratory failure. Overall, after 3 months of clinical follow-up, 82% of patients had favorable clinical outcomes (modified Rankin Score, [mRS] ≤2). Even in patients with initial poor clinical grading (Hunt–Hess grade 4–5), the 3-month favorable outcome rate still reached 70.8% (Table 1 and Table 2). The lower initial clinical grade is obvious effective to good outcome (*p* = 0.0207). A 92.3% favorable outcome rate was observed for patients with a low clinical grade (Hunt–Hess grade 1–3) and a 100% favorable outcome rate was observed for patients with a Hunt–Hess grade of 1–2. 60% (6/10) of patients with unconsciousness before angioplasty could regain consciousness; 70% of them could improve symptoms and 40% recover to achieve an excellent clinical outcome (Table 2).

**Table 1:** Characteristics of patients who accepted Scepter XC balloon angioplasty

	Total patients	H-H Gr 1–3	H-H Gr 4–5	Fisher Gr 1–2	Fisher Gr 3–4
Patients number	50	26 (52%)	24 (48%)	15 (30%)	35 (70%)
Age	50.0	48.0	50.9	48.5	50.4
Gender					
male	12	4	8	2	10
female	38	22	16	13	25
Symptomatic vasospasm	40	24 (60%)	16 (40%)	13 (32.5%)	27 (67.5%)
Unconscious without focal signs	10	2 (20%)	8 (80%)	2 (20%)	8 (80%)
Angioplasty Location:					
ICA MCA	25	9	16	8	17
M1	47	15	32	16	31
M2	35	10	25	14	21
M3 ACA	4	0	4	0	4
A1	8	3	5	3	5
A2	8	2	6	2	6
VA	7	1	6	0	7
BA	6	1	5	0	6
Need 2nd angioplasty	1 ^b^	0	1	0	1
Image Improving (%)	100%	100%	100%	100%	100%
Symptoms improving (%)	94% (47/50)	100%	87.5% (21/24)	100%	91.4% (32/35)
3 months good outcome (mRS < =2) (%)	82% (41/50)	92.3% (24/26) *p* = 0.0207	70.8% (17/24)	100% (15/15)	74.3% (26/35)
Mortality ^a^	2	0	2	0	2

*H-H* Hunt and Hess grade, *mRS* Modified Rankin scale

^a^ Mortality: 1 case of infarction with uncal herniation; 1 case of status epilepticus

^b^ Vasospasm occurred and angioplasty was performed on a different vessel

**Table 2:** Outcome of balloon angioplasty in unconscious patients and high clinical grading

Patients	Patients number	Angiography improving	Clinical improving	3 months mRS < =2
High clinical grading (H-H Gr. 4–5)	24	100%	21 (87.5%)	17 (70.8%)
Unconscious patients	10	100%	7 (70%)	4 (40%)

*H-H* Hunt and Hess grade, *mRS* Modified Rankin scale

## Discussion

### Early and combined endovascular procedures

Cerebral vasospasm remains a major cause of morbidity and mortality among patients after they survive initial SAH and undergo definitive aneurysm treatment. Of patients with aneurysmal SAH, 30 to 70% develop cerebral angiographic vasospasm, with death or permanent disability noted in 7 to 20% [1, 6, 7]. Younger age, poor presenting grade, and diffused subarachnoid clot are well-known risk factors for post-SAH vasospasm [2, 8, 22]. However, despite less favorable outcomes, patients with poor neurological status and even ischemic changes on CT scan, still appear to benefit from early endovascular therapy [6, 8]. Patients with poor consciousness status need not be excluded from this life-saving intervention [6, 8]. This is well demonstrated in our study of patients with significant vasospasm: the majority were of a younger age (mean age = 50 years), 68% presented with at least a Hunt–Hess grade of 3, and 70% had thick diffuse subarachnoid blood (Fisher grade > =3). In our study, 70.8% of patients with a high clinical grade (Hunt–Hess grade 4–5) could still attain a favorable outcome (mRS < =2); 60% (6/10) of patients with unconsciousness before angioplasty could regain consciousness and 40% recover to achieve an excellent clinical outcome. In these patients, the symptoms of vasospasm could not be detected easily and early. Therefore, early diagnosis by routine image study during the high-risk period of post-SAH vasospasm was necessary.

In the past, the endovascular approach was often required only in patients with symptoms that were refractory to medical management. However, no definite waiting time was suggested for “refractory.” In our study, 100% of angiographic and 94% of symptomatic vasospasms improved after angioplasty combined with balloon angioplasty and IA nimodipine, without any complications. These results compare favorably to those of other studies. In the recent literatures, the efficacy of mechanical balloon angioplasty was nearly 90 to 100%, associated clinical improvement rate of 60–75% and a complication rate of 5% [8, 17, 22–24]. For comatose and high clinical grading patients, the reports were rare and only 0 to 30% patients had good clinical result [8, 24]. Our results demonstrated that in patients with symptomatic or severe vasospasm, even with high clinical grade, neurosurgeons and neurointerventionalists should treat the condition earlier and endovascular therapy should be performed as soon as possible.

Simple IA vasodilator therapy is also effective but often transient, time insufficient, and requires multiple treatment sessions [12, 25]. Balloon angioplasty is suitable in larger vessels and has been reported as a relatively safe, effective, and durable procedure [12, 25]. Therefore, we recommend a combined procedure of balloon angioplasty in major vessels and continuous nimodipine infusion, which was effective for distal and diffuse vasospasm during the procedure. In our study, only one patient (2%) required a secondary session of angioplasty treatment. However, because the target vessel was different, the durability of balloon angioplasty remained apparent.

### Superiorities of scepter XC balloon for angioplasty

The Scepter XC balloon had favorable performance in the treatment of cerebral vasospasm in all consecutive patients without procedure-related complications in our series. The Scepter XC balloon was extremely trackable, facilitated safe, and was able to navigate distally into the intracranial circulation (the A2 and M3 segments). The Scepter XC balloon accommodates a larger 0.014-in. microwire, which provides significant stability to the balloon during navigation of tortuous vessels and distal advancement [13, 16]. Furthermore, during the balloon inflation, the 0.014-in. microwire provided increased stability, which resulted in less slippage along the vessel wall relative to the single lumen balloons that have been used previously. Last, we can use a single balloon for multiple segments with repeated inflation and deflation without the need to replace the balloon.

**Fig. 2:**
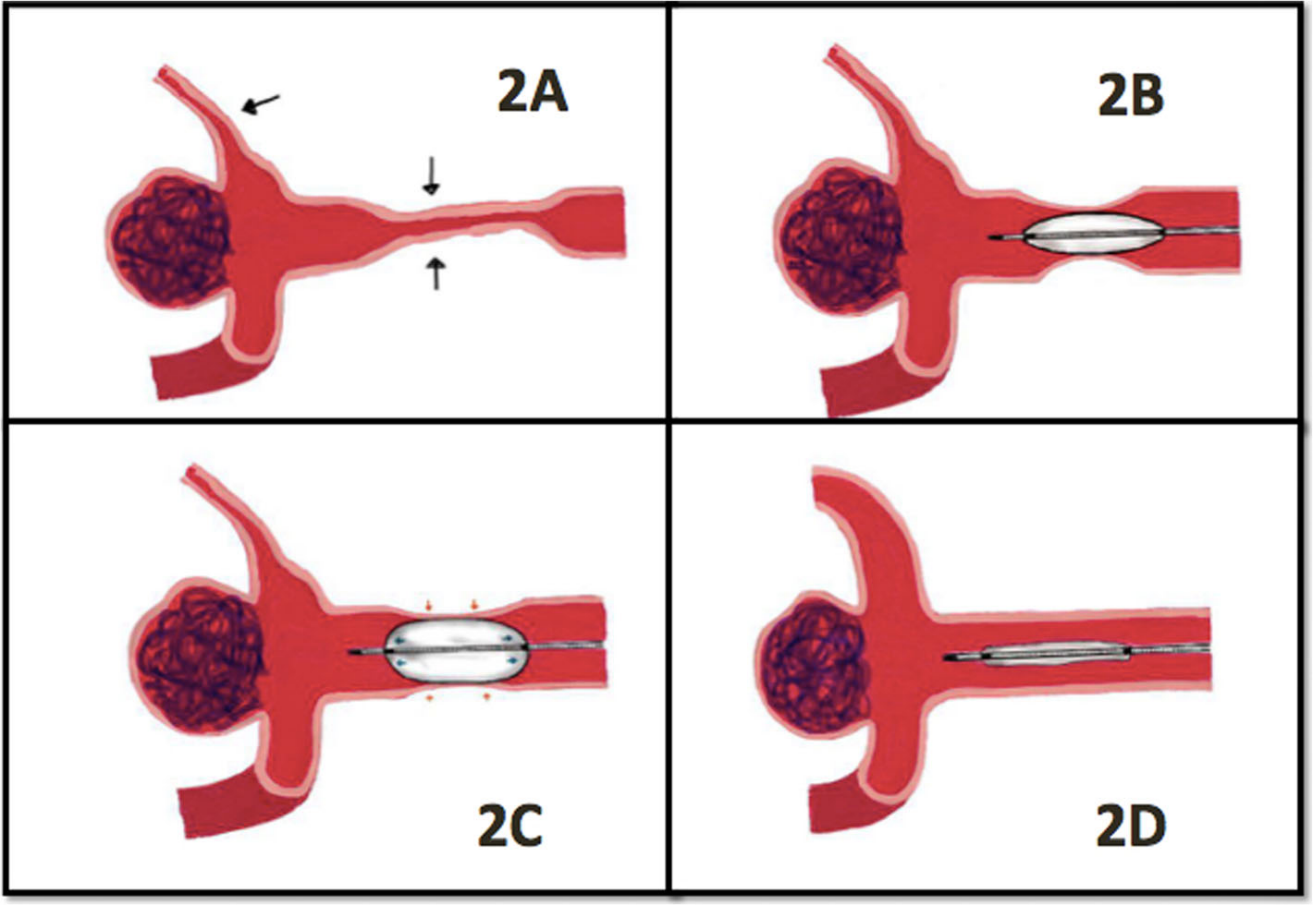
Demonstration of Scepter XC balloon angioplasty: **a** Middle cerebral artery vasospasm at M1 and M2 segments (black arrows). **b** Scepter XC balloon gradually inflated from the central area. **c** Even and gentle expansion of the balloon (blue arrows) along the vessel wall (red arrows). **d** Improvement of vasospasm after angioplasty at M1 and nimodipine treatment in M2 segment during the same session

**Fig. 3:**
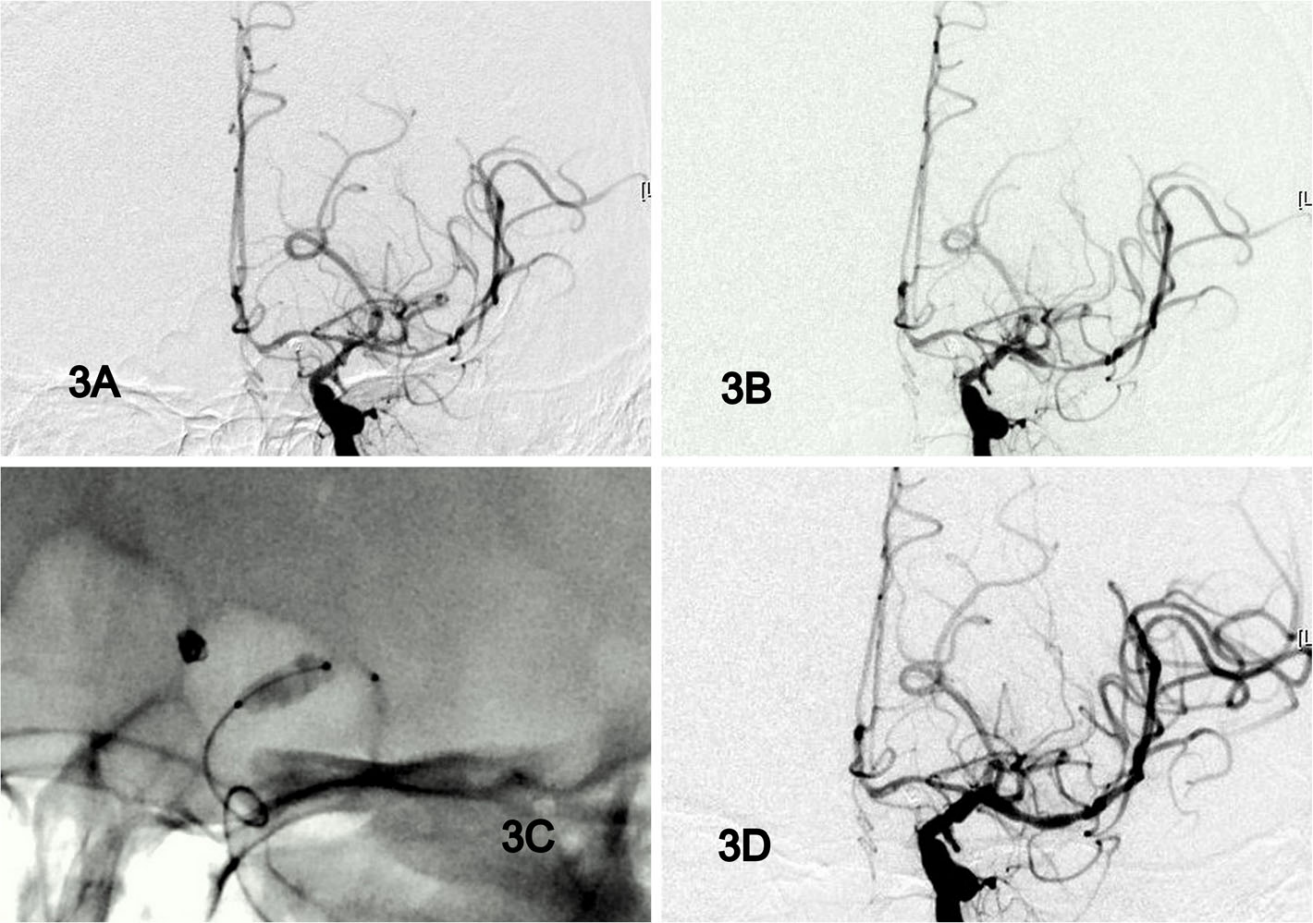
Case demonstration: **a** Diffused vasospasms at left MCA, M1, & M2 branches before angioplasty. **b** IA Nimodipine 30mins without angioplasty (**c**) Scepter XC balloon angioplasty from distal M1 to distal ICA. **c** After balloon angioplasty and simultaneous nimodipine infusion, the angiographic improvement is obviously

Studies have reported a complication rate of 1–4%; such complications are arterial rupture, dissection, and thrombus formation during angioplasty for vasospasm [10, 12]. Theoretically, the improved stability of inflation and the extra-compliant characteristic should be protective against arterial injury and rupture [15]. In our experience, during inflation, the extra-compliant design of the Scepter XC balloon tended to conform to the course of the vessel concurrent with radial expansion. The Scepter XC balloon was elliptically shaped at full inflation with two enhanced tips at both ends of the balloon. During inflation, the balloon gradually dilated from the central area and then bilaterally expanded evenly along the vessel wall (Fig. 2a, b). This conformation to the native vessel shape reflects the extra soft nature of the balloon and, in our opinion, leads to a more controllable and gentler balloon inflation. When the operator observes the balloon gradually expand near the two end markers of the balloon, the procedure could be ceased and deflation should be initiated; this characteristic of Scepter XC could prevent over-inflation, which causes vessel rupture (Fig. 2a & Fig. 3c). Generally, it takes less than 10 s to deflate the fully-inflated balloon in the diseased segment. Careful planning prior to endovascular treatment can reduce procedure time and lead to maximal improvement of cerebral vasospasm.

Moreover, simultaneous IA administration of nimodipine or other calcium channel blockers through the double lumen balloon catheter can augment the results of cerebral angioplasty, especially at distal circulation [9, 12]. In our experience, after a full angioplasty session, both target vessels, which were treated by balloon angioplasty, and distal vasospasms, treated by nimodipine, were improved simultaneously (Fig. 2d & Fig. 3d). After the diameter of proximal vessels was regained, more cerebral blood flow and more vasodilator effects could reach distal regions and increase cerebral perfusion. That may be why the effect of angioplasty is so durable and the functional outcome is so remarkable. In the Fig. 3, we demonstrated a case with symptomatic vasospasm (aphasia). Diffused vasospasms happened at left MCA, M1, & M2 branches before angioplasty (Fig. 3a). Initially, continuous IA nimodipine infusion was applied for over 30mins. The angiography revealed mild improving but still M1 stenosis (Fig. 3b); and her symptoms persisted. Scepter XC balloon angioplasty was performed with simultaneous nimodipine infusion during procedure. The angiographic result was remarkable (Fig. 3c) and the patient’s symptom recovered immediately after procedure.

### Limitation

The limitation of this study includes retrospective nature of the data analysis and absence of randomization between study groups. We were not able to distinguish the improvement from IA nimodipine alone, angioplasty, or combined effect; and to compare with diffident types of balloons and time interval between onsets of new deficits to intervention. However, we believed the result of the combination protocol with Scepter XC balloon angioplasty is extremely reliable because of high successful rate, good result, and low complications. Additionally, the clinical results may come from multivariable factors, such as hydrocephalus, surgical complications, infection, underlying comorbidities, and rehabilitations. Despite these factors, the effect might be minimized because of the single center with the same treatment protocol.

## Conclusion

Balloon angioplasty with Scepter XC balloon catheter is safe and effective for the treatment of cerebral vasospasm following SAH. Utilizing its extra-compliant characteristic could significantly improve the quality of angioplasty procedures. For all patients, even those with poor neurologic status, early endovascular intervention through combined strategy could improve clinical results.

## Data Availability

The datasets used and analyzed during the current study are available from the corresponding author on reasonable request.

## Abbreviations

SAH: Subarachnoid hemorrhage
CTA: Computed tomography angiography
DSA: Digital subtraction angiography
MCA: Middle cerebral artery
ICA: Internal carotid arteries
ACA: Anterior cerebral arteries
VA: Vertebral arteries
